# Mulberry Ethanol Extract and Rutin Protect Alcohol-Damaged GES-1 Cells by Inhibiting the MAPK Pathway

**DOI:** 10.3390/molecules27134266

**Published:** 2022-07-02

**Authors:** Tian-Yang Wu, Juan Liang, Jing-Ya Ai, Jing-Long Cui, Wei-Dong Huang, Yi-Lin You, Ji-Cheng Zhan

**Affiliations:** 1 Beijing Key Laboratory of Viticulture and Enology, College of Food Science and Nutritional Engineering, China Agricultural University, Tsinghua East Road 17, Haidian District, Beijing 100083, China; tianyangwu@cau.edu.cn (T.-Y.W.); Juanl2022@163.com (J.L.); aijingya@foxmail.com (J.-Y.A.); 2 Shandong ZISHEN Ecological Manor Co., Ltd., Shouguang 262700, China; cuijl2011@163.com

**Keywords:** mulberry ethanol extract, rutin, alcohol damage, gastric mucosal epithelial cells, oxidative stress

## Abstract

Mulberry extract has been proven to have the effect of resisting alcohol damage, but its mechanism is still unclear. In this study, the composition of mulberry ethanol extract (MBE) was identified by LC-MS/MS and the main components of MBE were ascertained by measuring. Gastric mucosal epithelial (GES-1) cells were used to elucidate the mechanism of MBE and rutin (the central part of MBE) helped protect against alcohol damage. The results revealed that phenolics accounted for the majority of MBE, accounting for 308.6 mg/g gallic acid equivalents and 108 substances were identified, including 37 flavonoids and 50 non-flavonoids. The treatment of 400 μg/mL MBE and 320 μM rutin reduced early cell apoptosis and the content of intracellular reactive oxygen species, malondialdehyde and increased glutathione. The qPCR results indicated that the MBE inhibits the expression of genes in the mitogen-activated protein kinase (MAPK) pathway, including p38, JNK, ERK and caspase-3; rutin inhibits the expression of p38 and caspase-3. Overall, MBE was able to reduce the oxidative stress of GES-1 cells and regulated apoptosis-related genes of the MAPK pathway. This study provides information for developing anti-ethanol injury drugs or functional foods.

## 1. Introduction

The increasing global consumption of alcoholic beverages has gained the attention of researchers to various diseases caused by alcohol. According to data published in *The Lancet*, between 1990 and 2010, alcohol consumption rose from sixth to third among all risk factors for disease, trailing only hypertension and smoking. Recent data found that alcohol consumption damaged stem cells’ DNA and caused mutations, possibly contributing to conditions such as cardiovascular disease (CVD) or cancer [1]. Once alcohol is administered, the stomach is the first organ to contact and absorb it. Heavy alcohol consumption is associated with an increased risk of mucosal damage. According to Ma and Liu, high-concentration alcohol can directly erode the gastric mucosa within 30 to 60 min, resulting in severe upper gastrointestinal bleeding and gastric mucosal lesions [2]. According to Oates and Hakkinen, alcohol can stimulate gastric peristalsis, which compresses blood vessels and reduces gastric mucosal blood flow [3].

Alcohol also causes inflammation in the gastric mucosa, increasing the content of reactive oxygen species (ROS), increasing the expression of inflammatory cytokines such as TNF-α and IL-1β and decreasing the expression of anti-inflammatory cytokines such as IL-10 [4]. In addition, Reddy proved that alcohol increased intracellular Ca^2+^ content and decreased membrane fluidity, leading to increased malondialdehyde (MDA) content in cells [5]. Yoo also found that alcohol causes oxidative damage to the gastric mucosa accompanied by gastric mucosal cell apoptosis and necrosis [6]. Cell apoptosis, oxidative stress and phenotypic lesions were always present when gastric mucosal epithelial (GES-1) cells were treated with alcohol to build an in vitro gastric mucosal injury model [7]. Existing studies show that drugs used to treat gastric ulcer diseases always cause side effects, such as histological damage and liver damage [8]. Many studies show that eating fruits and vegetables regularly effectively prevents gastric mucosal damage, which is attributed to their biologically active ingredients [9].

Mulberry is the fruit of the *Morus alba* L., a perennial woody plant of the genus Moraceae, with high nutritional value. The functional components of mulberry mainly include polyphenols, polysaccharides and alkaloids. Cho reported that mulberry has the effect of anti-thrombosis, anti-oxidation, anti-obesity, anti-inflammatory, anti-cancer and nerve protection without side effects [10]. The common flavonoids in mulberries are rutin, quercetin and catechin. Rutin was identified as the main antioxidant compound in mulberry and *Morus* sp. Rutin was proved to be effective in numerous diseases, including antioxidant, anti-diabetic, anti-hyperlipidemia and anti-obesity [11]. The phenolic substances in mulberry also have excellent antioxidant effects. The antioxidant activity of chlorogenic acid and its derivatives isolated from mulberry leaves helps prevent alcoholic steatohepatitis by reducing oxidative stress. Mulberry glycoside, a glycosylated stilbene found in abundance in the roots and branches of *Morus alba* L. (*Morus alba* Linn.), is beneficial in repairing alcoholic liver damage [12]. Cyanidin 3-sophoroside, cyanidin 3-glucoside, cyanidin 3-rutinoside, pelargonidin 3-glucoside and pelargonidin 3-rutinoside are the primary anthocyanins found in mulberry. In addition to bringing color to the fruit, it also has a robust radical scavenging ability. Mulberry anthocyanin extract can eliminate the excessive oxygen radicals produced in HepG2 cells that respond to oxidative stress, extend the lifespan of HepG2 cells by regulating Nrf-2 related signal pathways, and reduce the expression of TNF-α, IL-6, iNOS and NF-κB to reduce inflammation [13].

Mitogen-activated protein kinases (MAPKs) are serine-threonine kinases that mediate intracellular signal transduction processes related to various cell activities, including cell proliferation, differentiation, survival, death and transformation [14]. Arab reported that alcohol treatment increased the content of ROS in GES-1cells and induced oxidative stress, which may activate the MAPK signaling pathway and cause cell apoptosis [15]. The subdivision of the MAPK signaling pathway can also be divided into extracellular regulated protein kinase (ERK), p38 kinase (p38) and c-jun nh2-terminal kinase (JNK). The activation of p38 is often accompanied by the activation of JNK [16,17].

Mulberry extract is capable of resisting alcohol damage. However, its active ingredients and the mechanism have not been fully elucidated. Here, the composition of mulberry ethanol extract (MBE) was identified by using LC-MS/MS. GES-1 cells were used to explore the effect and mechanism of MBE and rutin on resisting alcohol damage.

## 2. Results

### 2.1. Composition Analysis and Identification of Phenolic Compounds in MBE

The content of the main components of the MBE was determined, including total sugar, total protein, oil, moisture, total anthocyanins, total phenols and rutin. The results are shown in Table 1, in which it can be seen that phenolic accounted for the main part. The total phenol reached 308.6 mg/g gallic acid equivalents, followed by the total anthocyanin (101 mg/g cyanidin-3-O-glucoside equivalents). Rutin is one of the main flavonols in mulberry [18]. It has a wide variety of biological functions, encompass scavenge radicals and relax blood vessels [19,20]. Rutin concentration was 19 mg/g as determined by the aluminum salt chelating color method. In addition to phenols, the total sugar content (65.9 mg/g), total protein content (28 mg/g), oil content (94 mg/g) and moisture content (84.3 mg/g) are also high.

**Figure 1:**
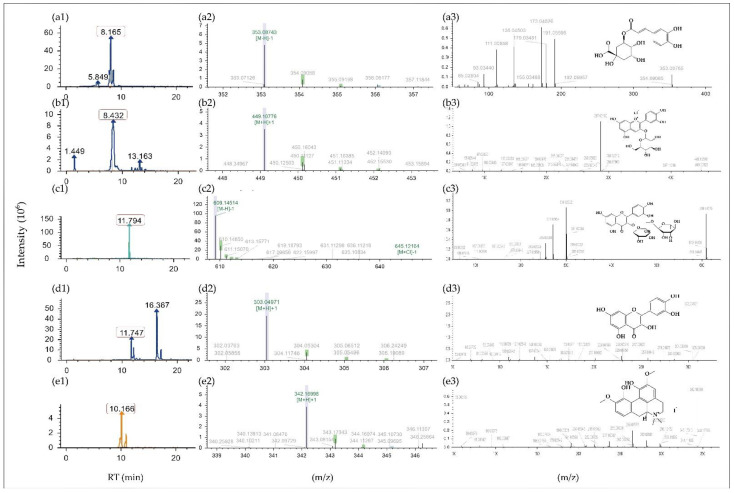
Retention time and mass spectrum of 5 examples: (**a1**–**a3**) Chlorogenic acid; (**b1**–**b3**) C3G; (**c1**–**c3**) Rutin; (**d1**–**d3**) Quercetin; (**e1**–**e3**) Magnoflorine.

The composition of MBE was investigated by LC-MS/MS (Table 2). The chromatograms, primary mass spectra, and secondary mass spectra of five examples are shown in Figure 1. In this work, 108 substances were identified in MBE, which can be classified by 87 phenolic, including 37 flavonoids (14 flavones, 11 flavonols, 7 flavanols, 1 isoflavones and 4 anthocyanines) and 50 non-flavonoids (5 chalcones, 2 resveratrol, 22 terpenoids, 16 phenolic acids and 5 other phenols). In addition, we also identified 5 amino acids, 2 carbohydrates, 3 alkaloids and 11 kinds of other substances. The findings revealed that phenolics have a significant role in MBE, with phenolic and flavonoids trailing behind. Flavonols, which have a similar general formula to rutin, are abundant in a variety of flavonoids. Chlorogenic and ginkgolic acids, as well as their derivatives, are the most common phenolic acids. In addition, the research yielded 22 terpenoids, each of which has a different physiological activity.

Overall, the most abundant substance in MBE is phenolic, with 87 different types. However, the type of biological action the MBE’s abundant polyphenols have is unclear.

### 2.2. MBE and Rutin Reduced the Alcohol Damage to GES-1

GES-1 cells were exposed to various amounts of alcohol for varying lengths of time. The normal state of GES-1 cells can be shown to be spindle-shaped, comparable to the neuron structure in the brain, evenly distributed, without overlap or aggregation between cells. Alcohol at 200 mM did not produce considerable harm to cells after 1 h, but alcohol at 400 mM caused significant damage within 1 h. After the action of alcohol, the cells changed from the original spindle shape to the shrunken shape and fell off of the petri dish; a large number of dead cells could be seen agglomerated into clusters. When the same concentration of alcohol was used to treat cells for varying durations, or different concentrations of alcohol were used for the same length of time, the morphological changes in the cells and cellular damage became more visible as the duration or alcohol concentration increased. Almost all the cell morphology of GES-1 cells was changed after treatment with 800~1000 mM alcohol for 6 h. The test results showed that it is feasible to screen the initial concentration of alcohol by directly observing the cell state in the experiment.

**Figure 2:**
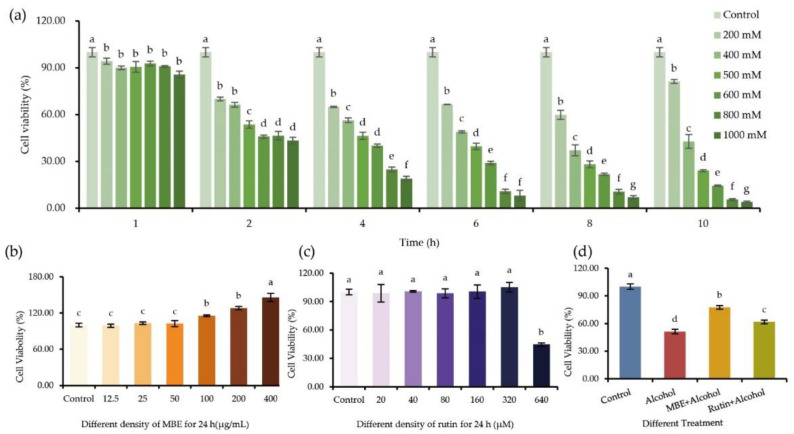
MBE and rutin can reduce the alcohol damage to GES-1. The cell viability was determined using MTT assay: (**a**) effect of different concentrations alcohol on GES-1 cells viability; (**b**) effect of MBE on GES-1 cell viability; (**c**) effect of rutin on GES-1 cell viability; (**d**) effect of MBE&rutin on alcohol injured-GES-1 cells viability; GES-1 cells were pretreated with 400 μg/mL MBE and 320 μM rutin for 24 h, then treat with 500 mM alcohol for 4 h. Different letters indicating significant differences labeled at the inhibitory activity at different compounds (*p* < 0.05).

The GES-1 cells were treated with different concentrations of alcohol for the corresponding time, and the cell viability was calculated by measuring the absorbance value by the MTT method, as shown in Figure 2a. Compared with the control group, alcohol in the concentration range of 200~1000 mM can cause noticeable damage to GES-1 cells, and 200 mM alcohol can damage the cells within 2 h. As concentration and action time rose, the damage became more visible. After 10 h of 200 mM alcohol, cell viability increased, related to alcohol volatilization and cell self-repair abilities. Typically, multiple quantities of alcohol are utilized for 2~5 h to screen the optimal best condition while building the alcohol-induced GES-1 cells injury model. The cell viability decreased to 46.43% after 4 h of exposure to 500 mM alcohol, which was significantly different from the control group and was relatively stable. Therefore, this study used exposure to 500 mM alcohol for 4 h to build alcohol-damaged GES-1 cells.

GES-1 cells were treated with several concentration gradients of MBE for 24 h to find the optimal circumstances. The MTT technique was used to determine cell viability after the treatment. Figure 2b depicts the effect of MBE on the viability of normal GES-1 cells. Although it did not promote proliferation, the MBE range concentration in 12.5~50 μg/mL did not have toxigenicity to cells. Meanwhile, MBE at 100–400 g/mL has a pro-proliferation effect on GES-1 cells and a significant difference (*p* < 0.05). As a result, the conditions for future tests were set at 400 g/mL MBE for 24 h.

The results of treating GES-1 cells for 24 h with different gradients of rutin and using the MTT method to detect cell viability are shown in Figure 2c. Rutin concentrations ranging from 20 μM to 320 μM had no harmful or substantial proliferative effects on GES-1 cells as compared to the control group. The rutin concentration of 640 μM is toxic to GES-1 cells, with cell viability dropping to around 40% after 24 h of treatment (*p* < 0.05). As a result, a rutin concentration of 320 μM was chosen for further research.

The GES-1 cells were pretreated with 400 μg/mL MBE and 320 μM rutin for 24 h, then discarded the culture medium and treated with 500 mM alcohol for 4 h before MTT detection was performed to calculate the cell viability. The results are shown in Figure 2d. Compared with the control group, treatment with 500 mM alcohol for 4 h caused significant damage to GES-1 cells and the cell viability dropped to under 50% (*p* < 0.05). After pretreatment with MBE, the cell viability of the group increased to about 77% (*p* < 0.05). The Rutin group also showed the same result; the cell viability reached 61%. Both substances have a specific protective effect on the damage caused by alcohol.

This study showed that exposure to 500 mM alcohol for 4 h was used as a model for alcohol-damaged GES-1 cells in this study. This alcohol concentration and processing time can establish a suitable cell model. Furthermore, pretreatment of GES-1 cells with 400 g/mL MBE or 320 M rutin for 24 h can significantly improve cell viability. However, it is still unclear in which way MBE and rutin enhance cell activity and resist alcohol damage in GES-1 cells. In follow-up experiments, we will explore anti-oxidation-related indicators to explore whether MBE and rutin can resist cell damage caused by alcohol by improving their antioxidant capacity.

### 2.3. MBE and Rutin Can Improve the Antioxidant Capacity of GES-1 Cells

The effects of MBE and rutin on alcohol-damaged GES-1 cells were evaluated based on intracellular ROS, MDA, SOD and GSH activity.

**Figure 3:**
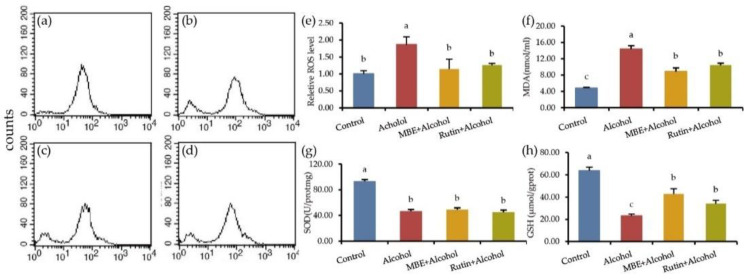
MBE and rutin can improve the antioxidant capacity of GES-1 cells. Panels (**a**–**d**) representative histogram of ROS analysis: (**a**) Control; (**b**) Alcohol, after treatment with 500 mM alcohol for 4 h; (**c**) MBE + Alcohol, GES-1 cells were pretreated with 400 μg/mL MBE for 24 h, then treat it with 500 mM alcohol for 4 h; (**d**) Rutin + Alcohol, GES-1 cells were pretreated with 320 μM rutin for 24 h, then treat it with 500 mM alcohol for 4 h; (**e**) the quantitative data of ROS in alcohol-damaged GES-1 cells; (**f**) effect of MBE and rutin on MDA in alcohol-damaged GES-1 cells; (**g**) effect of MBE and rutin on SOD in alcohol-damaged GES-1 cells; (**h**) effect of MBE and rutin on GSH in alcohol-damaged GES-1 cells. Different letters indicating significant differences labeled at the inhibitory activity at different compounds (*p* < 0.05).

GES-1 cells were pretreated with MBE and rutin for 24 h, then treated with 500 mM alcohol for 4 h, loaded with the DCFH-DA probe and the relative content of intracellular ROS was calculated after detecting the fluorescence intensity with flow cytometry. The results are shown in Figure 3a–e. Compared with the control group, the relative content of ROS in the cells increased significantly, reaching 1.86 times. After rutin pretreatment and alcohol treatment, the relative content of ROS in the cells decreased to 1.14 times and 1.2 times, compared with the alcohol group, there was a significant difference (*p* < 0.05). These results indicate that MBE and rutin can scavenge alcohol-damaged ROS. Additionally, it is worth noting that ROS is an essential regulator of cell function. High levels of ROS can damage cell lipids, DNA and proteins, while low levels of ROS have proliferative effects on cells [21,22].

MDA is the product of lipid peroxidation; its content will increase after oxidative stress. This test detected the MDA content in GES-1 cells after alcohol treatment. The results are shown in Figure 3f. As shown, the MDA content of the control group was 4.81 nmol/mgprot, which reached 14.37 nmol/mgprot after alcohol treatment. Compared with the control group, the content of intracellular MDA increased significantly after alcohol treatment (*p* < 0.05). The content of MDA of the MBE group was 8.83 nmol/mgprot and 10.46 nmol/mgprot of the rutin group. Compared with the alcohol group, MBE and rutin pretreatment can significantly reduce the intracellular MDA content (*p* < 0.05).

SOD is an essential antioxidant enzyme that can scavenge radicals to protect the body and reduce oxidative damage. After processing the cells according to the test requirements, they extract the protein and detect the SOD activity of the cells. The results are shown in Figure 3g. The SOD activity in the control group was 93.63 U/mgprot; after alcohol treatment, it was 46.93 U/mgprot. It showed that alcohol can significantly reduce SOD activity in the cells, which was quite different from the control group (*p* < 0.05). The SOD activity of the MBE group was 48.87 U/mgprot and the Rutin group’s SOD content was 40.05 U/mgprot. The MBE group and the rutin group did not increase the SOD activity. There was no significant difference between the alcohol group with MBE and the rutin group.

GSH is an essential antioxidant in cells that can scavenge oxygen radicals, a small molecule peptide composed of three amino acids. Since GSH is closely related to oxidative stress, this test detected the content of GSH in GES-1 cells after alcohol treatment. The results are shown in Figure 3h. The GSH content in the control group was 63.68 μmol/gprot. After alcohol treatment, it dropped to 23.04 μmol/gprot. Compared to the control group, 500 mM alcohol could significantly reduce the content of GSH in GES-1 cells (*p* < 0.05). The content of GSH was 42.85 μmol/gprot after MBE treated for 24 h and the GSH content was 34.09 μmol/gprot in the rutin group. The results show that MBE and rutin can significantly increase intracellular GSH (*p* < 0.05). It showed that MBE and rutin have a particular protective effect on the oxidative damage caused by alcohol.

Alcohol treatment caused oxidative stress in GES-1 cells, increased the content of intracellular ROS and MDA and decreased the content of GSH and SOD. After MBE and rutin treatment, the content of ROS and MDA significantly reduced, while the range of GSH increased. The results showed that MBE has excellent antioxidant capacity, which is related to the rich polyphenols such as rutin.

### 2.4. MBE and Rutin Can Reduce Alcohol-Damaged GES-1 Cell Apoptosis

**Figure 4:**
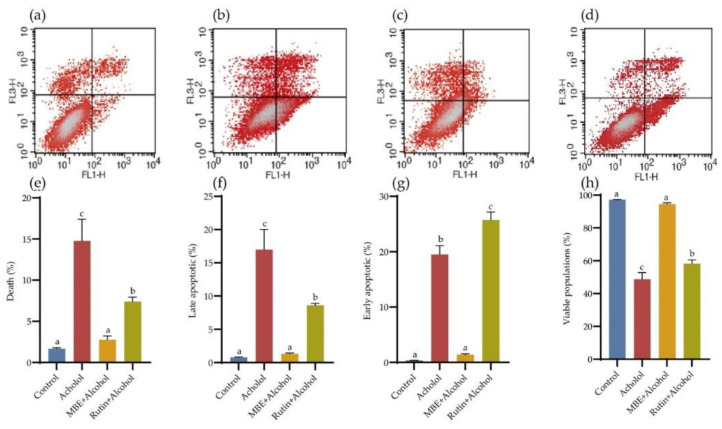
MBE and rutin can reduce alcohol-damaged GES-1 cell apoptosis. Flow cytometry was used to detect cell apoptosis. (**a**) Control; (**b**) Alcohol; (**c**) MBE + Alcohol, GES-1 cells were pretreated with 400 μg/mL MBE for 24 h, then treated with 500 mM alcohol for 4 h; (**d**) Rutin + Alcohol, GES-1 cells were pretreated with 320 μM rutin for 24 h, then treat with 500 mM alcohol for 4 h; (**e**–**h**) Effects of MBE and rutin on apoptosis of GES-1 cells, Compared with Control group. Different letters indicating significant differences labeled at the inhibitory activity at different compounds (*p* < 0.05).

Flow cytometry was used to detect cell apoptosis. The results are shown in Figure 4. The lower-left corner of the box represents the number of normal cells, the lower right corner represents the number of early apoptotic cells, the upper right corner represents the number of late apoptotic cells, and the upper left corner represents the number of necrotic cells. Under normal circumstances, the number of apoptotic GES-1 cells is minimal and the visible populations are 97.18 ± 0.36%. Alcohol treatment can significantly increase the apoptosis rate and the visible populations is only 48.75 ± 7.02%. The test results show that MBE and rutin can significantly reduce alcohol-induced apoptosis (*p* < 0.05). After MBE pretreatment for 24 h, the apoptosis rate can be reduced to 2.75 ± 0.823%, and the rutin group can be reduced to 7.40 ± 0.93%. The inhibitory effect of MBE on cell apoptosis is significant. The result of rutin is mainly manifested in delaying the occurrence of apoptosis.

The above experimental results showed that MBE and rutin have good anti-oxidation and anti-apoptosis abilities, but the metabolic pathway of their effects remains unclear. In the next experiment, we determine the critical signal pathway and genes to explore the possible mechanism of MBE and rutin against alcohol damage in GES-1 cells.

### 2.5. MBE and Rutin May Regulate the Antioxidant Capacity of GES-1 Cells by Regulating the Expression of MAPK Pathway Related Genes

**Figure 5:**
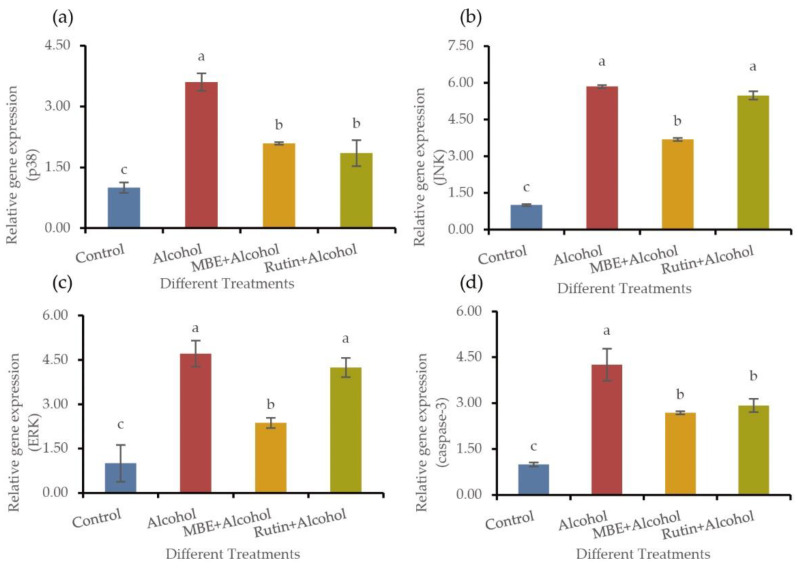
MBE and rutin may regulate the antioxidant capacity of GES-1 cells by regulating the expression of MAPK pathway related genes. (**a**) p38 relative gene expression; (**b**) JNK relative gene expression; (**c**) ERK relative gene expression; (**d**) caspase-3 relative gene expression. Different letters indicating significant differences labeled at the inhibitory activity at different compounds (*p* < 0.05).

The MAPK signal pathway may be involved in alcohol-induced gastric mucosal cell apoptosis. Three circuits can mediate the MAPK signal pathway: p38, JNK and ERK. We employed the fluorescence quantification method for the experiment to extract the RNA reverse transcription from the treated GES-1 cells and assess the expression of MAPK-associated genes. The results showed that MAPK might be the signaling pathway of alcohol-induced gastric mucosal apoptosis. Intragastric alcohol increased the phosphorylation of the MAPK pathway. As shown in Figure 5, the MAPK pathway can be mediated by p38, JNK and ERK. After processing GES-1 cells according to the experimental requirements, RNA reverse transcription and the qPCR of MAPK related genes were performed. In comparison, alcohol treatment can activate the expression of p38 (Figure 5a), JNK (Figure 5b) and ERK (Figure 5c) related to the MAPK pathway and eventually, cell apoptosis occurs caused caspase-3 (Figure 5d) expression (*p* < 0.05). MBE and rutin pretreated GES-1 cells for 24 h can significantly reduce the presence of the critical apoptosis caspase-3 (*p* < 0.05). In addition, MBE can considerably reduce the expression of p38, JNK and ERK in the MAPK pathway (*p* < 0.05). While rutin only decreased the expression of p38 (*p* < 0.05), but did not significantly affect JNK and ERK, which indicated that MBE might play a protective role through the MAPK pathway mediated by p38, JNK and ERK, while rutin may only activate the MAPK pathway through p38.

Based on the qPCR results, we can speculate that MBE improves the resistance of GES-1 cells to cell oxidation and apoptosis caused by alcohol damage by regulating p38, JNK and ERK. In this process, the rich polyphenols in MBE, such as rutin, played an important role. Polyphenols such as rutin in MBE will likely improve cell oxidation and apoptosis by regulating p38 in the MAPK pathway.

## 3. Discussion

Mulberry contains various compounds with high nutritional value, including phenolic acids, flavonoids, alkaloids and other biologically active compounds. Research on the functional components of mulberry has attracted many researchers from all over the world. At present, the extraction methods for the practical parts of mulberry and its trunk, root bark and leaves include solid–liquid extraction (SLE), microwave-assisted extraction (MAE), ultrasonic-assisted extraction (UAE), enzyme-assisted extraction (EAE) and pressurized liquid extraction (PLE) [11]. After comparing these extraction methods, it is found that traditional LE, MAE and PLE have poor selectivity, resulting in relatively large amounts of unwanted components being extracted, which will seriously affect subsequent separation and purification, and SPF is popular because of its simplicity, speed and high purity of the extracted samples. The methods can be selected for separation and purification by column chromatography, macroporous resin adsorption (MRA), silica gel chromatography (SGC) or ion-exchange chromatography (IEC) [23]. IEC is not only a controllable, high-selectivity and high recovery rate method, but it is also economically applied to large-scale industrial purification of biologically active substances.

This study showed that the main component in mulberry extracts was phenolic. Baba analyzed the components of five kinds of mulberry with 70% ethanol extracts with HPLC and found that the total phenol of gallic acid is 959.9~2570.4 μg/g [24], which is far lower than the 308.6 mg/g we measured. A study has shown that the total phenolics, flavonoids and anthocyanins in the freeze-dried powder of mulberry were 23.0 mg/g gallic acid equivalents, 3.9 mg/g rutin equivalents and 0.87 mg/g cyanidin-3-glucoside equivalents. The major flavonol in mulberry powder was rutin (0.43 mg/g), followed by morin (0.16 mg/g), quercetin (0.01 mg/g) and myricetin (0.01 mg/g) [25]. It can be seen that rutin is a more common polyphenolic in mulberry. However, the content of phenolic substances is less than the results determined in this study, which may be because of the different extraction methods. In this experiment, 95% ethanol was used for extraction, XAD-7 macroporous resin was used for purification, while Bae only used 70% ethanol for extraction. In addition, it may also be caused by the inconsistency of mulberry varieties, maturity and origin.

The composition of fruit, stems or roots is different, and some substances exist specifically. Ethanol can extract Morinin and Sanxinin from mulberry bark [26]. Mulberry glycosides can be obtained from mulberry bark using enzyme-assisted extraction and the macroporous resin method [27]. In addition, LC-MS/MS method was used to analyze the composition of mulberry leaf ethanolic extract. Eleven compounds were identified [28]. However, due to the composition differences between different parts of the *Morus alba L.*, this study could not characterize the alkaloids present in the two common mulberries (DNJ and mulberry leaf alkaloids).

The existing articles failed to report on the types of MBE systematically; they only summarized most of them. Qin isolated cyanidin 3-O-glucoside, cyanidin 3-O-rutinoside, pelargonidin 3-O-glucoside and pelargonidin 3-O-rutinoside with UV-Visible spectroscopy from the mulberry grown in Shaanxi, China [29]. Du identified three flavonoids (cyanidin 3-O-β-D-galactopyranoside, cyanidin 3-O-β-D-glucopyranoside and cyanidin 7-O-β-D-glucopyranoside) in Hangzhou native mulberry [30]. Some experimental results showed that 25 phenolic compounds from mulberry had been isolated [31]. However, in this work, 108 substances were identified in MBE, which can be classified into 87 kinds of phenolics, including 37 flavonoids and 50 non-flavonoids, far more than the results obtained in previous studies. However, the disadvantage of this experiment is that no standard products were used for comparison, which will be done in our subsequent work.

In cell experiments, different concentrations of alcohol were used to treat GES-1 cells to build an alcohol damage model. Under an optical microscope, results showed that a high concentration of alcohol treatment caused cells to shrink and become smaller and rounded. The number of cells floating in the base increased after culture [32]. The same phenomenon was found when the model was built in our study. Observation of the cell status directly is helpful for preliminary judgment of the screening concentration. Generally, cells were treated with different concentrations of alcohol for 2~5 h to select the optimal concentration and duration to build an alcohol-damaged model of the GES-1 cell injury model. A study showed that treating GES-1 cells with 400 mM for 6 h decreased the cell inhibition rate by 20% [33]. In this work, the optimal concentration and optimal duration of action were explored with the cell viability falling to about 50% as the modeling standard, and it was found that 500 mM of alcohol (a volume concentration of about 3%) can meet the modeling requirements after 4 h. The modeling conditions in different articles are different because alcohol is volatile and may be affected by alcohol purity, cell generation, cell state and culture conditions. However, a volume concentration of alcohol in the range of 2~8%, treated for 2~6 h, is commonly used. Liver cells were treated with 500 μM alcohol for 24 h, causing oxidative stress, increasing the content of intracellular ROS and MDA and decreasing the range of GSH and SOD [34], the same as in this study.

The MAPK signal pathway is also essential in the mitochondrial way of apoptosis. The MAPK pathway transduction axis contains at least three components: MKKK, MKK and MAPK. MKKK phosphorylates and activates MKK, thereby phosphorylating and activating MAPK. MAPK is composed of the extracellular ERK, p38 and JNK. Each of these enzymes has several isotypes: ERK1 to ERK8; p38-α, -β, -γ and -δ; JNK1 to JNK3, so the MAPK signaling pathway can also be divided into ERK, P38 and JNK, among which the activation of the p38 pathway is often accompanied by the activation of the JNK pathway [16,17]. The JNK and p38 pathways are activated by pro-inflammatory cytokines such as TNF-α and IL-1β. In the ERK signaling pathway, ERK1 and ERK2 (ERK1/2) are started by MEK1/2. MEK1/2 is activated by Raf, such as A-Raf, B-Raf or Raf-1. Raf-1 is activated by the small GTPases Ras, which is mediated by the receptor tyrosine kinase (RTK)-Grb2-SOS signal axis [35]. Camellia extract has similar composition to MBE, they both contain many anti-oxidant flavonoids, phenolic acid and other phenolic substances. A study showed camellia extract inhibited the phosphorylation of MAPK in the gastric mucosa cells [36]. Overall, this study showed that alcohol caused apoptosis of GES-1cells and altered the expression of genes related to the MAPK pathway. MBE and rutin (the main components of MBE) can inhibit this phosphorylation, thereby reducing cell apoptosis. In this study, the results will be complete if the determination of p38, JNK, ERK and caspase-3 protein expression can be increased. In addition, if knockout cells such as p38, JNK, ERK and caspase-3 are used in the experiment, the results can be more convincing.

## 4. Materials and Methods

### 4.1. Chemicals and Reagents

Cyanidin-3-oxy-glucoside, gallic acid and rutin were purchased from Sigma Aldrich (St. Louis, MI, USA). Trypsin, antibody and DMEM medium were purchased from Gibco (Grand Island, NY, USA). HPLC grade methanol, acetonitrile and formic acid were purchased from Merck (Darmstadt, Germany). Fetal bovine serum (FBS) was purchased from Tianhang Biotechnology Co., Ltd. (Zhejiang, China). Methyl thiazolyl tetrazolium (MTT), dimethyl sulfoxide (DMSO), ROS test kit and the protein concentration test kit was purchased from Beyotime Biotech Inc (Shanghai, China). MDA, glutathione (GSH) and superoxide dismutase (SOD) test kits were purchased from Nanjing Jiancheng Bioengineering Institute (Nanjing, China). The apoptosis test kit was purchased from Transgen Biotech (Beijing, China).

### 4.2. Determination of Ethanol Extract from Mulberry by LC-MS/MS

The mulberry was purchased from Xichang (Sichuan, China). Extraction was carried out using acetonitrile. Ultrasonic extraction for 100 kHz 30 min, then centrifuged at 12,000× *g* for 10 min, the supernatant was vacuum freeze-dried. The 75% methanol was used to dissolve the dried sample powder. The solution was centrifuged at 12,000× *g* for 10 min. The obtained upper phase was transferred to a liquid phase vial and stored at −20 °C. For stability considerations, samples were analyzed within 24 h.

Ultimate 3000 UPLC system (Thermo Fisher, Waltham, MA, USA), which was coupled with high-resolution mass spectrometry, QTRAP source (H-ESI, MS/MS) and a Waters HSS T3 column (100 mm × 1.8 mm, 2.5 μm) was used. Reagents: A: 0.1% formic acid (FA), B: acetonitrile (ACN), flow rate 0.3 mL/min, injection volume 2 μL, gradient: 0 min, 10% B; 1 min, 10% B; 10 min, 70% B; 10.5 min, 90% B; 12 min, 90% B; 12.1 min, 10% B, 40 min, 10% B. Heated-electrospray ionization (H-ESI) with negative polarity and positive polarity (3500 V) were used. The source temperature was 350 °C. The mass analyzer operates in the full mass-ddMS2 mode.

### 4.3. Determination of Main Components of Mulberry Ethanol Extract

Total sugar: The total sugar content in MBE (glucose equivalents) was determined by the anthrone sulfuric acid method [37]. The standard curve is made with glucose and the regression equation is y = 0.0041x−0.03 (R^2^ = 0.9996), unit: mg/g.

Protein: The protein content in MBE was determined by the Kjeldahl method [38] with a slight modification.

Oil: The oil content in MBE was determined by Soxhlet extraction [39] with a minor modification.

Moisture: MBE is dried at 103 ± 2 °C for 24 h, the weight loss of the sample is the moisture content.

Total phenol: The Folin–Ciocalteu colorimetric method [40] was used to determine the content of total phenols in the ethanol extract of mulberry (gallic acid equivalents) and the standard curve was made with gallic acid as the standard product. The regression equation was obtained as: y = 0.0012x + 0.1195 (R² = 0.9876), unit: mg/g.

Total anthocyanins: The content of total anthocyanins in MBE was determined by the pH differential method [41]. Absorb a certain amount of MBE solution and dilute it with hydrochloric acid sodium chloride buffer with pH = 1.0 and acetic acid sodium acetate buffer with pH = 4.5. Then, the absorbance of the two pH diluents was measured at 510 nm and 700 nm, respectively. The calculation formula is as follows:

A = pH1.0(A510 nm-A700 nm)-pH4.5(A510 nm-A700 nm)(1)

(2) $$\mathrm{Total}\;\mathrm{anthocyanins}\;(\mathrm{mg}/L)=\frac{\left(A\times \mathrm{MW}\times \mathrm{DF}\times 1000\right)}{\left(\varepsilon \times 1\right)}$$

Note: A is the absorbance of the sample; MW is the relative molecular weight of cyanidin-3-glucoside (484.82 mg/mol); ε is the molar absorption coefficient 24,825 M^−1^ cm^−1^; DF is the dilution multiple of the sample.

Rutin: The aluminum salt chelation colorimetric method was used to determine the content of rutin in the MBE and the standard curve was made with the standard rutin and the regression equation was obtained as y = 0.0052x + 0.0046 (R^2^ = 0.9945), unit: mg/g.

### 4.4. Cell Culture

GES-1 cells were donated by researcher Aibo Wu (Shanghai Institute for Biological Sciences, Chinese Academy of Sciences). GES-1 cells were routinely cultured in DMEM supplemented with 10% FBS and 1% penicillin and streptomycin (PS). Cells were maintained in a humidified incubator with 5% CO_2_ at 37 °C.

The concentration of MBE and rutin is 400 mg/mL, prepared with DMSO solution. Dilute with serum-free medium to 4000 μg/mL, filter sterilize and store at −20 °C for later use. Dilute to the required concentration during the test. The control group was treated with a serum-free medium containing 0.1% DMSO.

### 4.5. MTT Assay Detected Cell Viability

GES-1 cells were plated into 96-well plates at a density of 5 × 10^3^ cells/well. After cells were attached to the wall, replaced the culture medium with different treatments for 24 h. Culture solutions were removed and replaced by a new serum-free culture medium with MTT solution (5.0 mg/mL in PBS) for 3 h at 37 °C.

Remove the culture, add 150 μL DMSO, shake at a low speed for 10 min at 37 °C and measure the absorbance (A) value at 492 nm. Viability assays were performed using three independent experiments.

(3) $$\mathrm{Cell}\;\mathrm{vability}=\frac{\left(\mathrm{mean}\;\mathrm{OD}\;\mathrm{in}\;\mathrm{test}\;\mathrm{wells}-\;\mathrm{mean}\;\mathrm{OD}\;\mathrm{in}\;\mathrm{cell}\;\mathrm{free}\;\mathrm{wells}\right)}{\left(\mathrm{mean}\;\mathrm{OD}\;\mathrm{in}\;\mathrm{control}\;\mathrm{wells}-\;\mathrm{mean}\;\mathrm{OD}\;\mathrm{in}\;\mathrm{cell}\;\mathrm{free}\;\mathrm{wells}\right)}\times 100\%$$

### 4.6. Apoptosis Was Detected by the Annexin V Method

The apoptosis effect on GES-1 cells was studied using an Annexin V-FITC/PI Apoptosis Detection Kit (Beyotime, China) with a few modifications. Briefly, GES-1 cells were plated into 6-well plates at a density of 1 × 10^4^ cells/well. Until the cells were grown to 80% adherent, the cells were washed and collected with PBS at 4 °C, 1000 rpm, for 5 min. Cells were resuspended with 100 μL pre-chilled 1× Annexin V Bingding Buffer; then, 5 μL Annexin V-FITC and PI were added, mixed gently and allowed to react at room temperature for 15 min in the dark. A total of 400 μL pre-cooled 1× Annexin V binding buffer was added, mixed gently, placd on ice away from light and detected with BD FACS Lyric flow cytometer (BD Biosciences, San Jose, CA, USA) within 1 h. BD FACSDiva Software (Version 8.0.2) was used. Finally, fluorescence corresponding to the cell viability, apoptosis and necrosis of the harvested cells was subsequently analyzed.

### 4.7. Determination of Reactive Oxygen Species (ROS)

Cells were resuspended with 10 μmol/L DCFH-DA (diluted by serum-free culture medium), at a concentration of 1~20 × 10^6^ cells /mL for 20 min at 37 °C. The cells were washed with serum-free cell culture solution 3 times to remove the DCFH-DA that did not enter the cells fully. Then, detection took place by flow cytometry using 488 nm excitation and 525 nm emission wavelength. The fluorescence intensity of the control group was recorded as 100%, and the ratio of the fluorescence intensity of the other groups to the control group was the relative ROS level.

### 4.8. Determination of SOD, MDA and GSH

#### 4.8.1. Cell Protein Sample Collection

The treated cells were collected, a certain amount of Ripa lysate containing enzyme preparation was added into each well, these were lysed on ice for 2 min, then a small amount of liquid nitrogen was poured, and the cell sample was scraped off with a cell scraper and collected in the enzyme inactivated EP tube. This was then centrifuged at 4 °C for 15 min at 12,000 rpm. The supernatant was absorbed and stored at −80 °C for standby protein concentration determination.

#### 4.8.2. Determination of the Protein Concentration in the Sample 

The protein concentration was determined in the sample according to the instructions of the Pierce BCA protein detection kit 23225.

Kits A001-3-2, A003-4-1 and A005-1-2 were used for the determination of SOD, MDA and GHS, respectively. The experiment was conducted according to the instructions.

### 4.9. Quantitative Real-Time PCR (qPCR) Analysis

Total RNA was extracted using TRIzol^TM^ reagent (Invitrogen), according to the manufacturer’s instructions. Reverse transcription of the total RNA (2.5 μg) was performed with a high-capacity cDNA reverse transcription kit (Promega Biotech Co., Ltd., Madison, WI, USA). qPCR was run in triplicate for each sample and analyzed in a LightCycler 480 real-time PCR system (Roche). The oligonucleotides are shown in Table 3. The results were normalized to the internal control *β-actin*. The expression of genes related to the mitochondrial pathway of apoptosis (p38, JNK, ERK, caspase-3).

### 4.10. Statistical Analysis

All data reported in this paper are expressed as the means ± SEs. The data were evaluated by one-way ANOVA followed by Duncan’s significant difference test. All statistics and analyses of data were performed by SPSS version 25.

## 5. Conclusions

In this study, we first systematically classified and summarized the components of the MBE and found that the MBE and its main component rutin can reduce the damage caused by alcohol to GES-1 cells. The results suggest that MBE may play a role in eliminating ROS, reducing oxidative stress, and inhibiting the phosphorilation in MAPK pathway by inhibiting the expression of p38, JNK and ERK. Rutin may only exert this effect by inhibiting the expression of p38 in the MAPK pathway. However, this study still has some limitations, and in vivo experiments are still needed to prove the functions of MBE and rutin. Accordingly, it provides a theoretical reference for the development of MBE and its rutin and other polyphenols to prevent alcohol damage or make functional foods.

## Figures and Tables

**Table 1:** Composition analysis of MBE.

Composition	Quantification (mg/g)
Carbohydratesugar	65.9 ± 4.82
Protein	28 ± 1.69
Fats	94 ± 1.72
Water	84.3 ± 2.98
Anthocyanins (cyanidin-3-glucoside)	101.4 ± 8.14
Phenols (gallic acid)	308.6 ± 23.51
Rutin	19 ± 0.63

**Table 2:** The qualitative composition of MBE.

Category		N	RT	Accurate Mass	Molecular Ion [M−H]−/[M + H]+	Polarity	Molecular Formula	Putative ID	Fragment Ions [M−H]−/[M + H]+
Polyphenols	Flavones	1	8.139	448.1006	449.1082	+	C21H20O11	Cynaroside	287.05484
		2	12.778	594.1585	593.1509	−	C27H30O15	Kaempferol-3-O-gulcorhamnoside	285.03983/111.00861
		3	12.778	594.1585	595.1653	−	C27H30O15	Lonicerin	285.03983/111.00861
		4	12.980	624.1690	623.1616	−	C28H32O16	Isorhamnetin-3-O-nehesperidine	111.00859/314.04282
		5	12.980	624.1690	623.1615	−	C28H32O16	Narcissoside	111.00859/315.05096
		6	13.339	448.1006	447.0930	+	C21H20O11	Kaempferol-7-O-β-D-glucopyranoside	285.03970/447.09253
		7	16.301	286.0477	285.0403	−	C15H10O6	Luteolin	133.02939
		8	18.111	270.0528	269.0457	−	C15H10O5	Apigenin	269.04526
		9	18.873	316.0583	315.0510	−	C16H12O7	Eupafolin	300.02695
		10	22.143	552.1057	551.0979	−	C31H20O10	Bilobetin	519.07239/551.09796
		11	22.810	254.0579	253.0504	−	C15H10O4	Chrysin	63.02395/143.05013
		12	26.021	566.1213	567.1284	+	C32H22O10	Isoginkgetin	135.04411
		13	26.021	566.1213	567.1284	+	C32H22O10	Ginkgetin	135.04411/567.12842
		14	30.097	580.1370	581.1437	+	C33H24O10	Sciadopitysin	135.04413
	Flavonols	15	11.747	302.0427	301.0352	+	C15H10O7	Quercetin	151.00360/178.99858
		16	11.747	302.0427	301.0354	+	C15H10O7	Morin	151.00363
		17	11.794	610.1534	609.1467	−	C27H30O16	Rutin	300.02722/271.02454
		18	12.054	464.0955	463.0881	−	C21H20O12	Hyperoside	301.03476
		19	12.330	304.0583	303.0507	−	C15H12O7	Taxifolin	125.02422/285.04004
		20	12.778	594.1585	593.1509	−	C27H30O15	Kaempferol-3-O-rutinoside	285.03983/111.00861
		21	13.339	448.1006	447.0928	−	C21H20O11	Quercitrin	300.02713/301.03433
		22	13.339	448.1006	447.0928	−	C21H20O11	Quercetin 7-rhamnoside	300.02713/301.03433
		23	14.074	318.0376	317.0300	−	C15H10O8	Myricetin	137.02414/151.00339
		24	18.528	286.0477	285.0404	−	C15H10O6	Kaempferol	285.04016
		25	18.873	316.0583	315.0508	−	C16H12O7	Isorhamnetin	300.02695
	Flavanols	26	8.078	290.0790	289.0715	−	C15H14O6	Epicatechin	80.96500
		27	8.078	290.0790	289.0717	−	C15H14O6	(+)−Catechin hydrate	80.96500
		28	8.078	290.0790	289.0717	−	C15H14O6	Cianidanol	80.96500
		29	12.054	464.0955	463.0880	−	C21H20O12	Isoquercitrin	301.03476
		30	15.867	256.0736	255.0657	−	C15H12O4	Liquiritigenin	119.05003
		31	16.139	288.0634	287.0558	−	C15H12O6	Eriodictyol	135.04501/151.00346
		32	28.617	324.1362	323.1284	−	C20H20O4	Isobavachin	119.05003
	Isoflavones	33	16.425	284.0685	285.0756	+	C16H12O5	Calycosin	114.96126/225.05447
	Anthocyanines	34	6.941	340.0794	339.0717	−	C15H16O9	Esculin	177.0191
		35	8.139	448.1006	449.1078	+	C21H20O11	Cyanidin-3-O-glucoside chloride	287.05484
	Anthocyanidins	36	11.707	192.0423	193.0495	+	C10H8O4	Isoscopoletin	133.02841/178.02605
		37	11.707	192.0423	193.0496	+	C10H8O4	Scopoletin	133.02841/178.02605
	Chalcones	38	14.605	436.1370	435.1290	−	C21H24O10	Phloridzin	167.03477/273.07651
		39	14.605	436.1370	435.1290	−	C21H24O10	Trilobatin	167.03477/273.07651
		40	18.154	272.0685	271.0612	−	C15H12O5	Naringenin Chalcone	119.05000/151.00345
		41	18.154	272.0685	271.0611	−	C15H12O5	Naringenin Chalcone	119.05000/151.00345
		42	28.617	324.1362	323.1284	−	C20H20O4	Isobavachalcone	119.05006
Polyphenols	Resveratrol	43	8.139	448.1006	449.1078	+	C21H20O11	Astragalin	287.05484
		44	17.757	228.0786	229.0856	+	C14H12O3	Resveratrol	107.04910/135.04399/183.08055
	Terpenoids	45	12.054	326.1002	325.0928	−	C15H18O8	Bilobalide	163.11269
		46	12.720	440.1319	439.1242	−	C20H24O11	Ginkgolide C	125.02421/38313443
		47	16.025	408.1420	409.1483	+	C20H24O9	Ginkgolide A	345.13315
		48	16.150	424.1370	423.1294	−	C20H24O10	Ginkgolide B	113.02425/367.13931
		49	17.481	448.2309	493.2287	−	C21H36O10	Atractyloside A	59.01380/447.22342
		50	20.238	406.1264	405.1191	−	C20H22O9	Ginkgolide K	72.99297
		51	22.993	244.1099	245.1171	+	C15H16O3	Linderalactone	199.11176
		52	30.420	384.2301	385.2364	+	C24H32O4	Resibufogenin	109.02851/275.20062
		53	30.568	472.3553	471.3477	−	C30H48O4	Echinocystic acid	407.33185/471.34732
		54	30.691	470.3396	471.3471	+	C30H46O4	18 β-Glycyrrhetintic acid	189.16389/235.16933/317.21124
		55	31.275	454.3447	455.3516	+	C30H46O3	Ursolic acid	205.15874
		56	31.320	302.2246	303.2316	+	C20H30O2	Abietic acid	257.22626
		57	32.329	424.3705	425.3775	+	C30H48O	Lupenone	95.08546/109.10110
		58	33.739	472.3553	473.3625	+	C30H48O4	Maslinic acid	203.17943/409.34622/427.35706
		59	37.369	454.3447	455.3519	+	C30H46O3	Wilforlide A	205.15874
		60	37.369	234.1620	235.1691	+	C15H22O2	Curcumenol	189.16383/217.15863
		61	37.369	234.1620	235.1694	+	C15H22O2	Artemisinic acid	189.16383/217.15884
		62	37.369	454.3447	455.3520	+	C30H46O3	Oleanonic acid	205.15874
		63	37.369	454.3447	455.3502	+	C30H46O3	Liquidambaric acid	205.15874
Polyphenols		64	37.369	454.3447	455.3515	+	C30H46O3	β-Elemonic acid	205.15874
		65	37.369	456.3603	457.3668	+	C30H48O3	Ursolic acid	411.362
		66	41.687	440.3654	441.3733	+	C30H48O2	Roburic acid	95.08572/109.10140
	Phenolic acid	67	1.368	192.0634	191.0561	−	C7H12O6	Quinic acid	85.02938
		68	4.549	154.0266	153.0193	−	C7H6O4	Protocatechuic acid	109.02932
		69	4.549	154.0266	153.0192	−	C7H6O4	Gentisic acid	109.02932
		70	8.457	180.0423	181.0491	+	C9H8O4	Caffeic acid	163.03886
		71	8.475	354.0951	353.0876	−	C16H18O9	Cryptochlorogenic acid	173.04517/191.05585
		72	8.475	354.0951	353.0879	−	C16H18O9	Chlorogenic acid	173.04517/191.05585
		73	8.475	354.0951	353.0878	−	C16H18O9	1-Caffeoylquinic acid	173.04517/191.05585
		74	13.916	516.1268	515.1193	−	C25H24O12	1,3-Dicaffeoylquinic acid	173.04526/353.08749
		75	13.916	516.1268	515.1192	−	C25H24O12	Isochlorogenic acid C	173.04526/353.08749
		76	13.916	516.1268	515.1187	−	C25H24O12	Isochlorogenic acid B	173.04526/353.08749
		77	13.916	516.1268	515.1186	−	C25H24O12	Isochlorogenic acid A	173.04526/353.08749
		78	15.867	256.0736	255.0657	−	C15H12O4	Isoliquiritigenin	119.05003
		79	17.266	208.0736	207.0664	−	C11H12O4	Ethyl Caffeate	135.04500/207.06589
		80	43.102	320.2351	319.2277	−	C20H32O3	Ginkgolic Acid (C13:0)	275.23758
		81	43.546	346.2508	345.2430	−	C22H34O3	Ginkgolic Acid C15:1	301.25305
		82	46.176	374.2821	373.2745	−	C24H38O3	Ginkgolic acid C17-1	329.28458
	Other Phenols	83	4.237	182.0579	183.0653	+	C9H10O4	3,5-Dimethoxy-4-hydroxybenzaldehyde	81.03349/123.04411
		84	6.970	138.0317	137.0242	−	C7H6O3	Protocatechualdehyde	137.02423
		85	9.459	492.1268	493.1342	+	C23H24O12	Aurantio-obtusin β-D-glucoside	331.08093
		86	16.425	284.0685	285.0756	+	C16H12O5	Emodin-3-methyl ether/Physcion	114.96126/270.05191
		87	38.380	178.0630	179.0702	+	C10H10O3	Ferulaldehyde	147.04407/161.05971
Non- polyphenols	Amino acid	88	1.239	176.0432	174.9559	−	C5H8N2O5	3-[(Carboxycarbonyl) amino]-L-alanine	118.96609/146.96106
		89	1.702	115.0633	116.0707	+	C5H9NO2	L-Proline	70.0652
		90	1.893	181.0738	182.0812	+	C9H11NO3	L-Tyrosine	91.04922/119.04924/136.07571
		91	2.198	131.0946	132.1021	+	C6H13NO2	L-Leucine	86.09642
		92	3.123	165.0789	166.0862	+	C9H11NO2	L-Phenylalanine	130.08076
	carbohydrate	93	1.358	342.1162	387.1141	−	C12H22O11	Lactose	89.02428/179.05602
		94	8.647	518.1636	517.1561	−	C22H30O14	Sibiricose A5	175.03986
	Alkaloids	95	1.385	137.0476	138.0551	+	C7H7NO2	Trigonelline HCl	94.06523
		96	10.152	341.1627	342.1701	+	C20H23NO4	(+)-Magnoflorine	58.06545/342.16980
		97	16.940	365.1627	366.1699	+	C22H23NO4	Dehydrocorydaline	59.04945/32214359
	Other Compounds	98	1.312	182.0790	181.0721	−	C6H14O6	Mannitol	71.01373/101.02425
		99	1.895	192.0270	191.0197	−	C6H8O7	Citric acid	111.00856
		100	2.996	126.0317	127.0389	+	C6H6O3	5-hydroxymethyl furfural	109.02843
		101	7.638	376.1370	375.1294	−	C16H24O10	Loganic acid	213.07661
		102	7.652	460.1217	459.1143	−	C19H24O13	Parishin E	111.00859
		103	16.087	264.1362	263.1286	−	C15H20O4	Abscisic acid	204.13876/219.13876
		104	17.757	228.0786	229.0858	+	C14H12O3	Demethoxyyangonin	81.03352/183.08055/211.07530
		105	25.065	278.2246	279.2318	+	C18H30O2	α-Linolenic acid	149.02338
		106	26.121	486.3345	487.3416	+	C30H46O5	Quillaic acid	187.1481
		107	28.617	324.1362	323.1284	−	C20H20O4	Bavachin A	119.05013
		108	35.288	460.3916	461.3990	+	C30H52O3	20(R)-Protopanaxadiol	119.08558

**Table 3:** Oligonucleotides.

GENE	PRIMER (5′-3′)	SOURCE
β-actin	F: CTCCATCCTGGCCTCGCTGT R: GCTGTCACCTTCACCGTTCC	Sangon Biotech
caspase-3	F: GTGAGGCGGTTGTAGAAGAGTT R: CTCACGGCCTGGGATTTCAA	Sangon Biotech
p38	F: CCCACCCATATCTGGAGCAG R: GCCCTTGTCCTGACAAATTTAAGA	Sangon Biotech
JNK	F: CTGAAGCAGAAGCTCCACCA R: GCTGCCCCCGTATAACTCC	Sangon Biotech
ERK	F: TCAGACTCCAAAGCCCTTGAC R: TCAGCCGCTCCTTAGGTAGG	Sangon Biotech

## Data Availability

Not applicable.

## Sample Availability

Samples of the compounds are not available from the authors.
